# The role of protein lactylation in brain health and disease: current advances and future directions

**DOI:** 10.1038/s41420-025-02408-w

**Published:** 2025-04-30

**Authors:** Mingrui Han, Wenfeng He, Wengen Zhu, Linjuan Guo

**Affiliations:** 1 Department of Medical Genetics, The Second Affiliated Hospital of Nanchang University, Nanchang, Jiangxi China; 2 Queen Mary school, medical department, Nanchang University, Nanchang, Jiangxi China; 3 Department of Cardiology, The First Affiliated Hospital of Sun Yat-Sen University, Guangzhou, China; 4 Department of Cardiology, Jiangxi Provincial People’s Hospital, The First Affiliated Hospital of Nanchang Medical College, Nanchang, Jiangxi China

**Keywords:** Neurological disorders, Epigenetics

## Abstract

Lactate, the end product of glycolysis, plays a crucial role in cellular signaling and metabolism. The discovery of lactylation, a novel post-translational modification, has uncovered the role of lactate in regulating diseases, especially in the brain. Lactylation connects genetic encoding with protein function, thereby influencing key biological processes. Increasing evidence supports lactate-mediated lactylation as a critical modulator in neurological disorders. This review offers an overview of lactate metabolism and lactylation, highlighting recent advances in understanding the regulatory enzymes of lactylation and their role in the central nervous system. We investigate the impact of lactylation on brain dysfunctions, including neurodegenerative diseases, cerebrovascular disorders, neuroinflammation, brain tumors, and psychiatric conditions. Moreover, we highlight the therapeutic potential of targeting lactylation in treating brain disorders and outline key research gaps and future directions needed to advance this promising field.

## Facts

- Lactate, a carboxylic acid, plays a significant role in cellular signaling and brain metabolism.
- Lactylation, is an emerging post-translational modification that adds lactyl groups to both histone and non-histone proteins, participating in a variety of pathophysiological processes.
- Lactylation not only alters gene expression levels but also impacts biological functions by modifying the physicochemical properties of proteins.
- Studies indicate that targeting lactylation modifications is an effective strategy for treating central nervous system diseases.

## Open questions

- Research on the regulatory enzymes of lactylation modifications in the central nervous system is currently limited. Are there specific enzymes that mediate lactylation in the brain?
- What are the potential molecular mechanisms linking lactylation to central nervous system diseases, and how can we utilize these biological processes to guide the development of new therapeutic strategies?
- What role does lactylation play in the pathophysiological processes of the central nervous system? A protector or a disruptor?

## Introduction

Brain diseases, such as neurodegenerative disorders, cerebrovascular conditions, and psychiatric illnesses, are major health challenges that are increasingly associated with metabolic dysfunctions [1]. Among these, the role of lactate has emerged as a key area of interest because of its role in cellular signaling and brain metabolism. Although traditionally viewed as a metabolic waste product linked to hypoxic conditions, the significance of lactate in various biological processes is now being reevaluated [2].

Lactylation, a post-translational modification (PTM) identified in 2019, introduces a novel mechanism by which lactate can modulate protein function and gene expression [3]. This discovery has opened new avenues for understanding how metabolic changes impact brain health and diseases. The regulation of lactylation involves specific enzymes, termed writers, erasers, and readers, which control the addition and removal of lactyl groups and thereby influence key cellular functions [4]. While the role of lactate in brain metabolism is well-studied, the impact of lactylation is still under-researched, particularly in relation to brain disorders.

Although prior studies have discussed the roles of lactylation in conditions such as gliomas [5], neurogenesis [6], and neurodegenerative diseases [6, 7], they often suffer from incomplete coverage of regulatory enzymes and outdated references. To bridge these gaps, this review offers an up-to-date overview of lactylation research in brain health by including the latest published studies. Furthermore, we systematically categorize lactylation regulatory enzymes for the first time to investigate their role in lactylation across various neurological diseases. We have summarized the pathophysiological processes of neurodegenerative diseases, cerebrovascular disorders, neuroinflammation, brain tumors, and psychiatric conditions, along with the mechanisms of lactylation modifications’ involvement. In conclusion, we determine that targeting lactylation possesses substantial therapeutic potential and significantly contributes to mitigating metabolic dysfunctions in neurological diseases.

## Lactate metabolism and its role in the brain

Under normal physiological conditions, glucose is converted to pyruvate in the cytoplasm and subsequently enters the tricarboxylic acid (TCA) cycle in the mitochondria, producing substantial energy, oxygen, and water for cellular activities. In hypoxic or other adverse environments, respiration proceeds anaerobically, converting pyruvate from glucose to lactate by lactate dehydrogenase (LDH) via anaerobic glycolysis [8]. At rest, the brain is responsible for 13% of the body’s lactate production and 8% of its utilization. During exercise, the brain’s lactate uptake increases significantly, consuming about 11% of the body’s lactate, underscoring the critical role of lactate metabolism in brain function and systemic homeostasis [9]. Even under aerobic conditions, cancer cells often prefer anaerobic glycolysis, known as the Warburg effect [8, 10], which produces energy more rapidly than oxidative phosphorylation (OXPHOS) and satisfies the increased demand for glycolytic intermediates, supporting rapid tumor growth and leading to poor clinical outcomes [8, 11, 12].

Lactate is cleared either by oxidizing to pyruvate for re-entry into the TCA cycle or by converting back to glucose in the liver and skeletal muscle via the Cori cycle [13, 14]. Lactate exists primarily in two enantiomeric forms: l-lactate and d-lactate [8]. l-lactate is produced directly by glycolysis, whereas d-lactate formation in eukaryotes occurs mainly through two pathways: the glyoxalase enzyme system and gut microbiota [13]. In the glyoxalase pathway, a branch of glycolysis, glucose is converted to methylglyoxal and subsequently processed by glyoxalase 1 (GLO1) and GLO2 into d-lactate and glutathione. Meanwhile, gut microbes, such as lactic acid bacteria, also convert glucose into d-lactate, which enters the bloodstream [13].

L-lactate, the predominant form in the human body and brain, is involved in various biological processes, such as regulating energy production and metabolic pathways [13, 14]. Lactate enters cells through monocarboxylate transporters (MCTs) on the cell membrane or via GPR81 receptor-mediated signaling pathways [14]. MCTs are extensively expressed throughout the brain [8, 14]. The intercellular lactate shuttle describes the transfer of lactate from glycolytic cells to surrounding cells. In the nervous system, the most extensively studied is the astrocyte-neuron lactate shuttle (ANLS) [7]. Astrocytes take up glucose from the bloodstream, store it as glycogen, and produce lactate via rapid glycolysis in response to heightened neuronal activity. This lactate is then transported via MCTs to the extracellular space, where neurons uptake it through MCTs, converting it to pyruvate and acetyl-CoA for ATP production via OXPHOS, which supports synaptic formation and neurotransmitter release [7, 15]. This evidence challenges the conventional view of lactate merely as a metabolic waste product. In the brain, lactate’s metabolic ecology and its spatial and temporal distribution are closely tied to cerebrovascular blood flow, neuronal excitation and neuroplasticity [16]. A significant outcome of the inflammatory pathology linked to cytotoxicity and hypoxia within the tumor microenvironment is an increase in local tissue lactate levels [17]. Recent research underscores the vital roles of lactate in enhancing memory impairments, alleviating neurological dysfunctions, and fostering neuronal repair and regeneration [8].

## Lactylation development and its mechanisms

### Development of lysine lactylation

PTMs alter the biochemical properties of specific amino acid residues in proteins, which changes their functions and regulates gene expression. The concept of enhanced lactate production was first described a century ago by the Warburg effect, which revealed that tumor cells rely on anaerobic glycolysis even in the presence of oxygen, providing them with numerous advantages. Subsequently, extensive research on lactate has led to the recognition that lactate is no longer considered a metabolic waste product, but rather plays a crucial role in various biological processes such as cellular metabolism and signaling [4]. In 2019, Professor Zhao Yingming’s team made a groundbreaking discovery, identifying lactyl-CoA-mediated histone L-lysine lactylation (Kla) in human breast cancer MCF-7 cells and its involvement in gene expression regulation. This modification was confirmed through immunoblotting and isotope labeling experiments, demonstrating that it originates from intracellular glycolysis [3].

Lactylation modifications exist in three distinct stereochemical forms: lysine l-lactylation (Kl-la), ε-(carboxyethyl)-lysine (Kce), and d-lactyl-lysine (Kd-la). Kl-la, the predominant form of lactylation mediated by lactyl-CoA, is tightly regulated by glucose concentration and glycolytic levels [18]. This novel modification type is present in various cellular compartments, and its extensive diversity in protein substrates is evidenced by its dual targeting of nuclear histones and cytoplasmic non-histones. Since 2019, histone lactylation has been extensively studied. Changes in chromatin structure affect gene expression, which in turn plays a decisive role in cellular function and fate. It has been confirmed to play a critical role in the development of various diseases, including cancer, neuropsychiatric disorders, and cardiovascular diseases [19]. For instance, H4 lysine 12 lactylation (H4K12la) induces smooth muscle cell senescence by promoting the expression of aging-related genes, thereby accelerating atherosclerosis [20]. As research progresses, non-histone targets have also been gradually identified, which exhibit a pronounced preference for enzymes involved in metabolic pathways [21]. Non-histone lactylation can influence the spatial localization, conformation, and other characteristics of target proteins, thereby regulating the stability of protein–protein interactions and ultimately altering their functional state. By constructing l-lactate sodium derivatives, numerous potential non-histone sites have been identified [22], further affirming their significance beyond histones. Notably, recent studies have for the first time revealed that guanosine triphosphate (GTP)-specific SCS (GTPSCS) and acetyl-CoA synthetase 2 (ACSS2) are responsible for synthesizing lactyl-CoA. Both of them participate in histone lactylation, with the assistance of P300 and KAT2A, respectively, playing a significant role in the development of gliomas [23, 24]. With this discovery, the synthesis mechanism of lactylation modification precursors has been elucidated, marking a new milestone in lactylation research. These studies position lactylation as a crucial player in the pathophysiological processes of various diseases, significantly expanding our understanding of the role of lactate in disease contexts.

### Enzyme-mediated lysine lactylation

The precise execution of enzyme-mediated lactylation requires the assistance of specialized tools. Writers, erasers, and readers are three pivotal regulatory enzymes in this process, managing the addition of lactylation modifications, the elimination of lactyl groups, and their spatial distribution. l-lactate, the substrate for lactylation, is supplied through two pathways: one is the conversion of extracellular glucose molecules into pyruvate through glycolysis, which is then catalyzed by LDH to form l-lactate; the other method involves direct transport of l-lactate into the cell via MCT. GTPSCS-mediated conversion of l-lactate to lactyl-CoA is considered a prerequisite for catalysis, as the writers cannot directly add the lactyl group from lactate to lysine residues [8, 23]. Recently, the discovery of new catalytic mechanisms of alanyl-tRNA synthetase 1 (AARS1) has shown that l-lactate can be converted into lactyl-AMP, which can also be utilized by writers, indicating that they are not entirely reliant on lactyl-CoA [25, 26]. Once the catalytic substrates are ready, writers, erasers, and readers are responsible for regulating the subsequent lactylation modification process (Supplemental Table 1).

#### Writers

Writers are responsible for adding lactylation modifications to both histone and non-histone proteins. Researchers have identified numerous enzymes that can act as lactylation architects. Most enzymes belong to three main classical histone acetyltransferase (ACT) family (HAT or KAT): p300/CREB-binding protein (CBP) family involving p300 and CBP, MYST family including MOF, HBO1, and Tip60 and Gcn5-related N-acetylase (GNAT) family involving GCN-5 and Yiac [27]. Other enzymes, such as AARS and alpha-tubulin ACT1 (ATAT1) have also been found to play a role. Current research indicates that the efficiency of most writers in adding lactyl groups depends on the intracellular levels of lactate synthesis and the ability of MCT to transport lactate into cells, using lactyl-CoA as the substrate. However, AARS is an exception, which relies on lactate-AMP as an intermediate to directly transfer lactyl group to the lysine residues of target proteins with the assistance of ATP [25, 26]

As the most well-studied writer, p300-catalyzed lactylation mainly promotes gene transcription [28–39] and maintains normal protein structure and functions [40–45]. In the central nervous system (CNS), P300 and its homologous protein CBP participate in the physiological processes of various diseases by mediating histone and non-histone lactylation. For example, pre-treatment with a small molecule inhibitor of P300 significantly reduced the level of lactylation in a mouse model of ischemic stroke, helping to maintain neuronal activity and alleviate ischemic brain injury [46]. In microglia, lactylation modifications act as a double-edged sword in their impact on abnormal brain pathological conditions. Retinal microglial cells undergo P300-mediated lactylation of YY1, activating microglial cells by promoting the transcription of certain inflammatory genes [38]. In addition, the promoting role of P300/CBP and its associated factors in the aging process of microglia in Alzheimer’s disease (AD) has also been observed. However, in certain cases, this epigenetic modification can induce the transition of microglia to the M2 repair phenotype when lactate levels are increased [47]. For glioblastoma multiforme (GBM), the P300-mediated writing process profoundly influences disease progression and treatment strategies. P300-mediated histone lactylation has been observed to be predominantly localized at transcription start sites, significantly driving the malignancy of the U87MG GBM cell line and promoting immunosuppression in the GBM microenvironment. The enhanced efficacy of GBM immunotherapy is attributed to the elimination of histone lactylation by P300 inhibitors [48]. Recent groundbreaking findings have identified GTPSCS as lactyl-CoA synthetase in the nucleus of glioma cells, promoting glioma progression through P300-mediated histone lactylation [23]. Thus, P300/CBP-mediated protein lactylation plays a crucial role in various brain diseases and brain cell activities.

Overall, the mechanisms by which P300 mediates lactylation modifications require further investigation and refinement. The process of gene expression regulation involves a complex regulatory network, including chromatin assembly and the transmission of genetic information, with histone chaperones being one of the key components [49]. Initially recognized in the cardiovascular field, ASF1A has been identified as a cofactor that assists P300 in catalyzing H3K18 lactylation [39]. This indicates that the P300-mediated lactylation process is unlikely to be isolated, and its regulatory factors warrant further study. In terms of disease treatment, regulating lactylation levels in pathological states presents a potential strategy. However, it is crucial to consider how to balance lactate concentration with the catalytic effects of P300. Among the determinants of the final lactylation modification levels, the “substrate” appears to be more critical than the “tool”. In conditions such as pancreatic ductal adenocarcinoma and heart failure, the effects caused by changes in lactate levels cannot be reversed merely by manipulating P300 [33, 41].

#### Erasers

The “eraser” refers to enzymes that remove lactylation modifications on lysine residues. Histone deacetylases (HDAC), common in mammals [33, 50], are de-lactylation enzymes that effectively remove Kl-la and Kd-la modifications. Mammalian deacetylases are divided into two major families: zinc-dependent HDAC 1-11, also known as Class I, II, and IV HDACs, and NAD-dependent sirtuins (SIRT) 1-7, categorized as Class III HDACs [27]. Initially, HDACs are generally believed to preferentially target histones, whereas sirtuins exhibit a notable preference for non-histone components in the cytoplasm or within the nucleus, exhibiting complementary functions [51, 52]. Nonetheless, sirtuins also play a role in the removal of histone lactylation at the cellular level. The potent de-lactylation activity of HDAC1-3, observed in both cellular and in vitro purified proteins, was first identified by Yruela et al., particularly regarding Kd-la [53]. While HDACs may more commonly erase Kl-la modifications, the removal of Kl-la compared to Kd-la might involve specific mechanisms and depend on the cross-linking of different substances within the nucleus [51].

The regulation of erasers is of great significance for the development and function of CNS. Inhibition of HDAC1-3 has been shown to modulate neuronal identity during murine neurodevelopment, activating transcription. H3K9 crotonylation (H3K9cr), H3K9 acetylation (H3K9ac) and H3K18la dynamically change in the genome during embryonic development, working together with other PTM modifications to determine chromatin accessibility and neuronal fate [54]. Additionally, the de-lactylation of H4K8 by SIRT2 has been shown to suppress neuroglioma cells [55]. HDACs and SIRTs, by removing lactylation modifications, regulate the levels of lactylation in the CNS together with the writers. The balance between these enzymes plays a crucial role in gene expression and protein stability in normal brain cells.

#### Readers

Readers are defined as enzymes that regulate and recognize lactylation modifications, with research in this area being largely unexplored. Current research on readers primarily focuses on their role in tumor progression, such as in colorectal cancer [31]and cervical cancer [56]. In the nervous system, the function of readers has also been elucidated. Evidence suggests that H4K8la levels of astrocytes in subarachnoid hemorrhage (SAH) are modulated by Bromodomain-containing protein 4 (BRD4). Silencing of BRD4 profoundly affects H4K8la levels, with immunoprecipitation experiments directly demonstrating their interaction [57]. Given BRD4’s capacity to engage with enhancers and histone acetylation marks, forming intricate interactions to exert robust regulatory effects on gene transcription, BRD4 may be a potential reader in this context [58, 59]. Readers, in collaboration with writers and erasers, collectively influence the dynamic balance of lactylation modifications in cells, playing a significant role in the regulation of cellular activities. Further studies are required to fill the gaps in this field.

### Non-enzymatic lysine lactylation

**Fig. 1:**
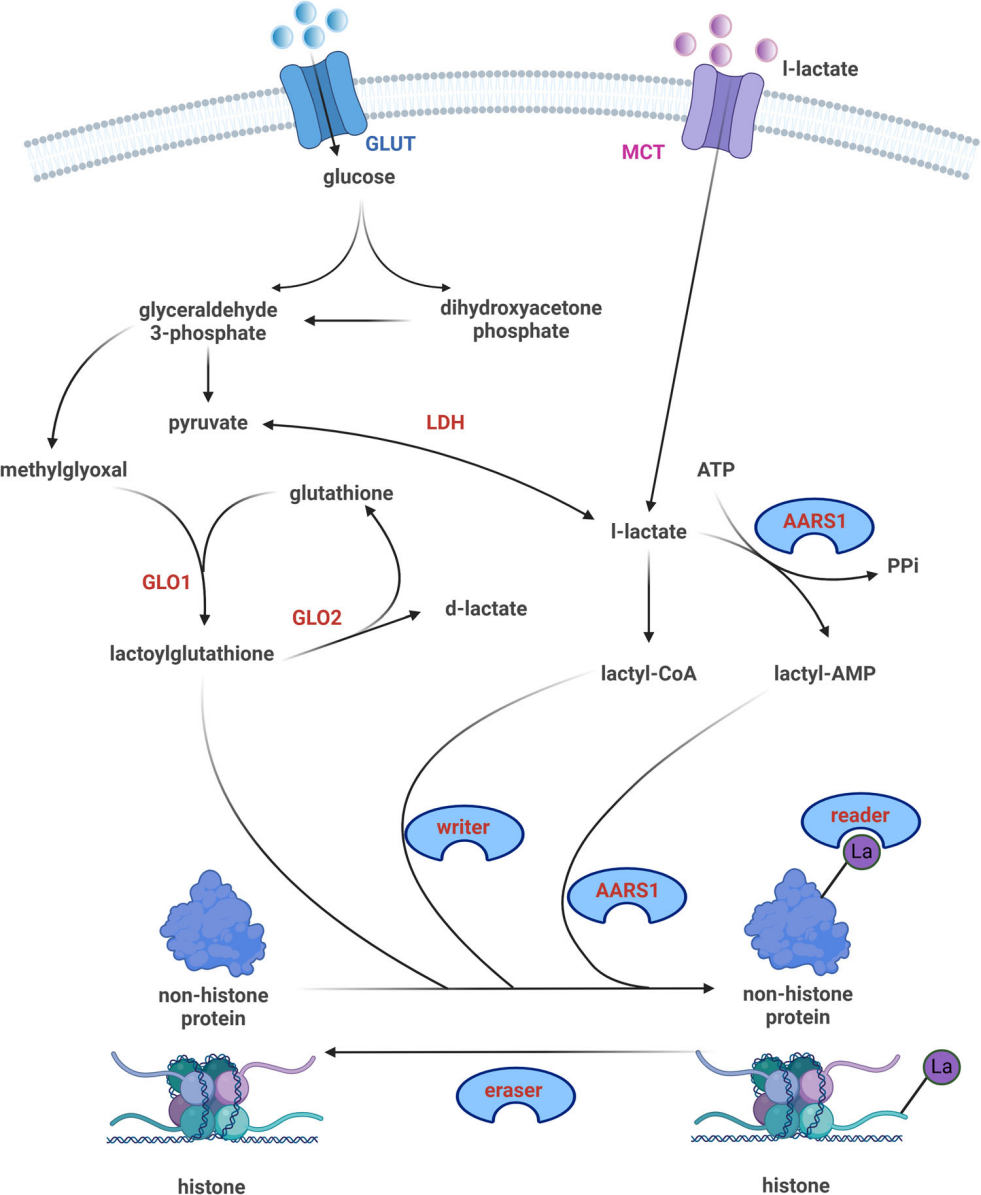
Lactate synthesis and regulatory mechanisms of lactylation. The lactate metabolism pathway and the regulatory mechanisms of lactylation are illustrated as shown. Lactylation modification targets include both histones and non-histones, and it is accomplished through two pathways: enzyme-catalyzed and non-enzyme-catalyzed reactions. The first pathway: l-lactate which is transported into the cell through MCT or synthesized from pyruvate is the primary substrate for enzyme-catalyzed lactylation. The catalytic process of lactylation by the writer requires the synthesis of lactyl-CoA, while AARS1 uses lactyl-AMP as an intermediate. The balance of lactylation modification is ultimately regulated by erasers and readers, with the former removing lactylation marks and the latter influencing the addition of lactyl groups. The second pathway: non-enzyme-catalyzed lactylation refers to the spontaneous interaction between lactoylglutathione and lysine residues, mainly regulated by GLO1 and GLO2. The substrate, lactoylglutathione, is derived from a specific metabolic pathway from glycolysis.

The non-enzymatic lactylation process involves the interaction of lactoylglutathione (LGSH) with lysine residues. The spontaneous incorporation of lactoylLys was initially validated by Gaffney et al. and this process is meticulously regulated by LGSH and GLO2 [60]. Compared to Kl-la, Kd-la is more prone to spontaneous lactyl group addition mediated by LGSH [11]. This is further supported by Zhang et al., who demonstrated that impairing the GLO pathway specifically disrupts the production of Kce and Kd-la, while the generation of lactyl-CoA appears to be positively correlated with Kl-la [18] (Fig. 1).

### Methodology in lactylation measurement and identification

The study of lactylation modifications involves various technical approaches and detection methods to thoroughly elucidate the complex biochemical metabolic pathways and regulatory mechanisms. Mass spectrometry (MS), which chemically cleaves proteins into peptide fragments for characteristic peak analysis, is one of the first high-sensitivity classical methods employed. In 2019, Zhang et al. conducted high-performance liquid chromatography (HPLC)–tandem mass spectrometric (MS/MS) analysis on peptides derived from histones in MCF-7 cells and surprisingly discovered a mass shift identical to that induced by the addition of a lactyl group to lysine residues [3]. The advantage of this method lies not only in its ability to confirm the presence of lysine modifications but also in its capacity to identify specific modification sites.

In addition to MS, the use of anti-lactylation antibodies represents another well-established detection method. Generally, antibodies are categorized into those that target all lactylation modifications, such as Pan-Kla, and those that target specific sites, such as anti-H3K18la. By specifically recognizing lactylation sites and combining this with immunofluorescence staining, it is possible to detect lactylation modifications on target proteins and is available to precise localization within tissues. When investigating the role of lactylation modifications in the tumorigenesis of ocular melanoma, researchers utilized Pan-Kla antibodies and DAPI to perform immunofluorescence staining on ocular melanoma tissues and normal melanocyte tissues. This approach clearly demonstrated an elevated level of lactylation in the ocular melanoma tissues [29].

Proteomics and bioinformatics analyses are often used in conjunction to identify candidate genes and explore the functions of lactylation in different cell types and tissues. By performing enrichment analysis on metabolic pathways and signaling pathways related to lactylation modifications in relevant databases, researchers can infer the effects of this PTM on target proteins. For instance, when Yao et al. aimed to investigate the critical role of lactylation in cerebral ischemia-reperfusion injury (CIRI), they first identified upregulated and downregulated lactylation sites among various proteins through proteomics. Subsequently, they explored the biological pathways involving differentially expressed proteins in the database, ultimately elucidating the potential impacts of lactylation modifications [61].

In the future, with the advancement of artificial intelligence (AI), the accuracy, speed, and scalability of identifying PTMs will continue to improve. AI algorithms, particularly machine learning models, can analyze large datasets to identify patterns and associations that traditional methods may struggle to detect. This capability will facilitate the more efficient discovery of lactylation sites, enhance our understanding of their functional roles in various diseases, and drive the development of new therapeutic strategies.

## Protein lactylation in brain health and diseases

### Neurodegenerative diseases

AD is characterized by pathological features including amyloid-β (Aβ) accumulation, tau protein aggregation, neuroinflammation, and age-related neuronal degeneration [62]. Compromised glucose metabolism is a key feature of AD, typically appearing as the second pathological change after Aβ deposition [63, 64]. This metabolic impairment in neurons directly disrupts OXPHOS, resulting in elevated lactate levels and increased lactylation [65]. Astrocytes, through the ANLS, are pivotal in sustaining the normal physiological functioning of neurons [15]. The dysregulation of glycolysis in these cells has been identified as a significant initiator of neuronal impairments in AD [66]. Additionally, microglial and astrocytic inflammation can damage neurons, induce oxidative stress, and worsen AD progression [67]. Recent studies have established a close link between amyloid protein, which is associated with metabolic reprogramming and the acute inflammation of microglia, and the activation of glycolysis, which in turn directly stimulates microglial activation [68]. A metabolic shift from OXPHOS to glycolysis raises tissue lactate levels, thereby establishing a foundation for lactylation in AD. Lactylation-related genes such as ARID5B, SESN1, and XPA have been identified as pivotal regulators in AD pathology [69]. Recently, lactylation has garnered research interest for its potential link to AD.

Pan and colleagues [70] discovered that in the 5XFAD transgenic mouse model of AD, lactate-mediated H4K12la expression in the hippocampus shows a marked preference for microglia surrounding Aβ plaques. This process further leads to lactate accumulation through enrichment in the transcriptional start site of the glycolysis-related gene pyruvate kinase M2 (PKM2), highlighting the glycolysis/H4K12la/PKM2 feedback loop’s significant role in AD progression. The periodontal pathogen Porphyromonas gingivalis can express small RNAs (msRNAs) similar to microRNAs to modulate host gene expression. Macrophages transfected with msRNA P.G_45033 exhibit enhanced glycolysis levels increased histone Kla levels, and accumulation of Aβ in periodontal tissue that may cross the blood-brain barrier and enter the brain [71]. In addition, the reduced activity of a key TCA cycle enzyme, isocitrate dehydrogenase 3β (IDH3β), can cause lactate buildup and histone lactylation at sites like H4K12, H4K8, and H3K18. The transcription factor PAX6, a downstream target of IDH3β, negatively controls IDH3β expression, creating a positive feedback loop with IDH3β, lactate, lactylation, and PAX6 [65].

Glucose metabolism disruptions occur not only in pathology but also with aging, marked by metabolic abnormalities and bioenergetic dysregulation [72]. Thus, aging is closely linked to the onset of AD. Cognitive impairment in AD patients is closely associated with age-related genomic damage and mitochondrial dysfunction [73]. Recent research has extensively explored the link between lactylation and aging, beyond neurodegeneration, in conditions like intervertebral disc degeneration [74] and skin aging [75]. In AD research, Wei et al. have created mouse models like the aging FAD4T, AD-specific APP/PS1, and mature microglial Dox-BV2, showing that H3K18la enhances NFκB signaling by upregulating the expression of Rela (p65) and NFκB1 (p50) [76]. This process ultimately triggers aging-related events, including the secretion of senescence-associated secretory phenotype (SASP) factors like IL-6 and IL-8 by aging cells, which are strongly correlated with histopathological features [77]. Their findings underscore the critical role of the lactate/H3K18la/NFκB/IL-6 and IL-8 pathways in both aging and AD [76]. In addition, pan-Kla has been found to regulate Kmt2a gene transcription in the hippocampal tissue, potentially disrupting synaptic plasticity and affecting cognitive functions in mice, such as spatial exploration and memory, by altering synaptic protein expression [78]. Long-term intervention with lactate and high-intensity interval training (HIIT) in elderly mice showed an increase in the expression of several angiogenesis-associated pathway proteins in the hippocampus, enhancing mitochondrial activity and metabolism-related substances. This further illustrates the beneficial long-term effects of lactate and lactylation on enhancing brain neuroplasticity and slowing neurodegeneration [79]. It is noteworthy that the hippocampus, the brain region most closely associated with AD, exhibits Aβ accumulation in nearly all AD mouse models. The aforementioned studies further underscore its critical role in susceptibility to aging and AD progression, serving as a key memory controller [80, 81].

Microglia, key regulators of neuroinflammation, have been demonstrated to modulate AD progression through lactylation modifications. Notably, tau protein lactylation at K677 initiates ferroautophagy and ferroptosis through the MAPK pathway, leading to microglial activation and neuroinflammation [82]. Microglial autophagy, mediated by the lysosomal system, effectively clears Aβ. An imbalance in Aβ generation and clearance is a pathogenic factor in AD [83]. EPB41L4A-AS1 is a metabolism-related long non-coding RNA (lncRNA), that is thought to be closely associated with aging and AD progression due to its deficiency causing ATP synthesis defects [84]. In the EPB41L4A-AS1-specific knockout astrocyte cell line U251, Aβ_1-42_ addition led to significant impairment in Aβ_1-42_ clearance and expression of autophagy-related genes (ARGs). It is hypothesized that EPB41L4A-AS1 modulates the transcription of specific ARGs by regulating GCN5L2 lactylation enzyme activity, thus affecting Aβ removal [85].

**Fig. 2:**
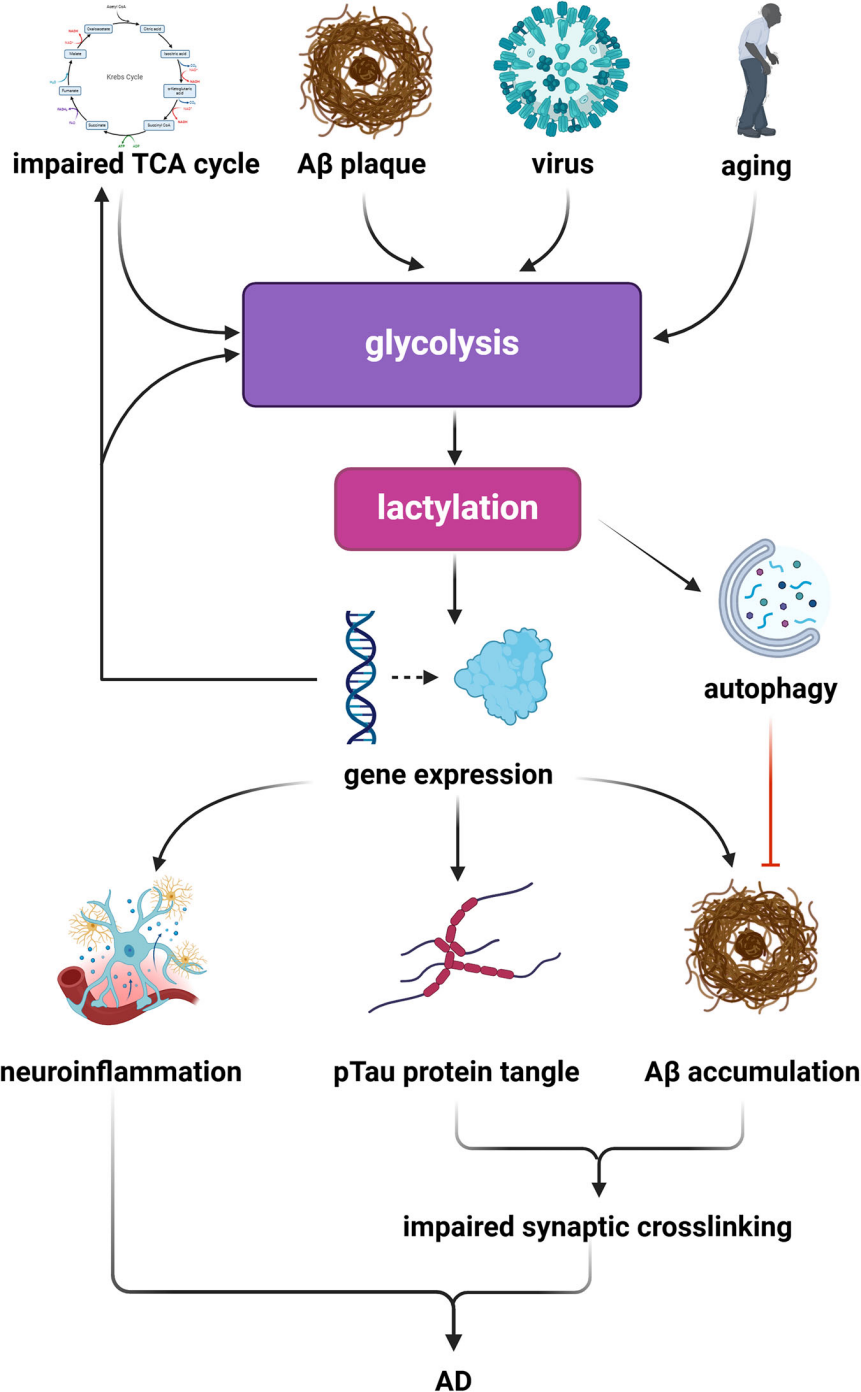
The regulatory role of lactylation in AD pathology. In AD, the enhancement of glycolytic metabolism is associated with various factors, such as dysregulation of glucose metabolism, accumulation of Aβ plaques, viral infections, and natural aging. Increased lactate production further regulates gene expression through lactylation modification, which can exacerbate neuroinflammation, pTau protein tangles, and Aβ plaque deposition. These pathological changes can impair synaptic connectivity and ultimately accelerate the progression of AD. It is important to note that, in certain instances, lactylation can promote the transcription of autophagy-related genes, playing a beneficial role in the clearance of Aβ plaque accumulation.

Promising therapeutic strategies can be provided from multiple perspectives. Considering the upstream and downstream associated proteins, inhibiting PKM2 might reduce abnormal microglial activation and inflammation, thereby improving regional neuronal health and enhancing spatial learning and memory in mice [70]. Similarly, upregulating IDH3β or downregulating PAX6 could prevent phosphorylated tau accumulation, restoring synaptic protein expression and ameliorating pathological symptoms in 5xFAD mice [65]. In addition, the role of physical exercise in brain function recovery and neuronal repair should not be overlooked [47, 79]. It is important to note that the role of lactylation in Alzheimer’s disease has not been fully elucidated, meaning this specific modification does not necessarily promote disease progression. Occasionally, lactylation can act as a protective response to neuroinflammation. In this process, exercise-induced lactate accumulation can trigger the “lactate clock,” promoting the transition of microglia to a reparative phenotype [47]. Further research is required to investigate the targeted cell types and specific stages of the disease process in which lactylation plays a role in AD (Fig. 2).

In addition to AD, lactate metabolism dysfunction and lactylation modifications are also associated with other neurodegenerative diseases. Parkinson’s disease (PD) is primarily characterized by motor symptoms, with the loss of dopaminergic neurons being a key feature [86]. Due to mitochondrial dysfunction and a lack of energy supply, cellular metabolism gradually shifts toward glycolysis, leading to lactate accumulation and further dopaminergic neuron damage [87]. Recent studies have shown that enhanced glycolytic turnover promotes microglial activation through H3K9 lactylation, which in turn triggers neuroinflammation and accelerates the progression of PD. The use of the glycolysis inhibitor 2-DG has been shown to alleviate the pathological condition in mice by reducing lactate accumulation, suggesting that targeting glucose metabolism may be a potential strategy for treating PD [88]. Multiple sclerosis (MS) is a chronic, immune-related neurodegenerative disease [89]. Increased aerobic glycolysis has been observed in newly diagnosed MS, and the extent of this increase correlates positively with the severity of the disease [90]. Studies have shown that patients with relapsing-remitting MS exhibit elevated lactate levels in their cerebrospinal fluid and monitoring this biomarker may become a powerful tool for early identification and prognosis assessment of the disease [91]. Although no research has yet established a direct link between lactylation modifications and MS, this area holds significant exploratory potential.

Overall, there is a significant and undeniable connection between lactylation modifications and neurodegenerative disorders. However, it is essential to recognize that AD, as a classic neurodegenerative condition, has a complex and multifaceted pathogenesis. A significant limitation of current studies on lactylation is their predominant reliance on mouse models that represent only specific pathological features, which do not fully reflect the actual conditions of patients’ brains. A new mouse model has been developed that simultaneously characterizes Aβ accumulation and tau protein aggregation [92]; however, a significant challenge remains in improving these models to better reflect real-life conditions. Moreover, the study of the localization of lactylation modifications in relation to disease pathology is extremely important. For example, Pan et al. reported elevated levels of Pan-Kla and H4K12la in the cortex and hippocampus of 5XFAD mice, showing significant co-localization with microglia [70]. In contrast, another study found that H3K18la levels were only elevated in the hippocampus [76]. Thus, it is essential to focus on the differences among various tissues. Furthermore, current studies indicate that lactylation can have both positive and inhibitory effects on the pathology of AD. We speculate that this phenomenon may be related to temporal factors and cell types. One possible explanation is that microglia possess specific mechanisms to sense intracellular lactate concentrations. The initial metabolic shifts in microglia tend to trigger neuroinflammation and phagocytic suppression [65, 76]. When abnormalities in the local microenvironment and further increases in lactate levels exceed a certain threshold, microglia may undergo a compensatory shift toward a repair phenotype through lactylation. Another possibility is that the involvement of astrocytes contributes to the recovery of cognitive function during physical activity. The elevated levels of glycolysis in astrocytes can enhance the neuronal energy supply via ANLS, thereby alleviating energy deficits and facilitating functional recovery.

### Cerebrovascular disorders

Cerebrovascular disorders (CVD) constitute a spectrum of disorders marked by pathological alterations in the cerebral vasculature. These changes result in functional impairments due to changes in hemodynamic forces, the structural integrity of the vascular walls, and other contributing factors [93]. Stroke, as the most common form of CVD, encompasses ischemic stroke (IS), hemorrhagic stroke, and the less frequently occurring SAH [94]. The IS is the leading killer affecting human health in CVD, characterized by reduced tissue oxygen availability, a shift in metabolic pathways towards glycolysis, and increased lactate concentration [95]. Additionally, elevated serum lactate is also an important marker for intracerebral hemorrhage (ICH) [96]. Beyond the optimal treatment timeframe for IS, the resumption of blood flow and oxygen delivery can precipitate additional tissue damage, a phenomenon termed CIRI. CIRI is closely associated with perturbations in reactive oxygen species (ROS), which stem from inflammatory reactions, excessive calcium levels, and mitochondrial impairment [97]. This injury manifests in various cellular damages, encompassing apoptosis [98], ferroptosis [99] and pyroptosis [100]. Lactate has traditionally been considered a parallel indicator of neuronal dysfunction. However, recent studies have found that moderate supplementation of lactate after ischemic injury and reperfusion is more effective than glucose in improving neurological outcomes [101]. Lactate has also been shown to stimulate angiogenesis and facilitate tissue repair subsequent to ICH [102]. Lactylation, which occurs in response to heightened lactate concentrations, is intricately connected with the pathogenesis and progression of CVD.

In ischemic CVD, there is an inseparable link between damage caused by IS and inflammation in the central nervous system. Within the brain, vascular dysfunction, pathological alterations in the cellular environment, and microcirculatory hypoxia frequently act as instigators of neuroinflammation, often interconnecting in a complex web [103–105]. The neuroinflammatory damage they induce is a key factor in the progression of IS. Timely termination of the inflammatory activation of microglia and astrocytes is a crucial prerequisite for tissue recovery and the improvement of neurological function [106, 107]. As important components of the innate immune system, cyclic GMP-AMP synthase (cGAS) and its downstream signaling protein stimulator of interferon genes (STING) are adept at swiftly detecting foreign danger signals in cells and initiating an inflammatory response [108]. In the brain, they are considered key mediators in regulating the inflammatory activity of microglia during the pathological process of IS [109]. Infants suffering from hypoxic-ischemic encephalopathy (HIE) demonstrate an accelerated rate of glycolytic turnover and a marked increase in cGAS expression. The lactylation modification at the K162 site of cGAS is a critical determinant in the polarization of microglia towards the M1 phenotype [110]. A groundbreaking study has unveiled that lactylation of the Lys131 residue at the N-terminus of cGAS by AARS2 in the wake of viral infection leads to a loss of DNA recognition capacity. This mechanism could underlie the immune suppression associated with elevated tissue lactate levels [111]. Similarly, A1 astrocyte activation, along with lactate-mediated lactylation, has been noted to occur in the early stages of IS. Proteins that have undergone lactylation are inclined to engage in the modulation of neuronal activity, which can exacerbate neuronal damage and lead to impaired brain function [46].

Neuroinflammation is a critical event following cerebral ischemia, with extensive inflammatory responses leading to cell damage after reperfusion. In this process, the role of lactylation is not only evident in mediating neuroinflammation but also instrumental in regulating various cellular death pathways. These modifications exert their influence by modulating the protein stability and altering the expression levels of specific mediators. High mobility group box 1 (HMGB1) serves as a key nuclear protein, representing a damage-associated molecular symbol when exists in extracellular environment. Its expression is regulated by various PTMs and has been shown to be closely related to pyroptosis [112]. Yao et al. found that in the CIRI model, increased LDHA-mediated H3K18 lactylation modification promotes the transcription of HMGB1. Specific knockdown of LDHA using SiRNA significantly reduces intracellular HMGB1 levels and prevents pyroptosis [113]. Nuclear receptor coactivator 4 (NCOA4) acts as a crucial receptor mediating the transport of ferritin cargo to the lysosome, making it an essential regulator of ferritinophagy [114]. The stability of the NCOA4 protein can be reinforced by lactylation at the K450 residue, which in turn triggers ferritinophagy and leads to ischemic stroke-related neurological dysfunction [115]. The cytoskeletal protein Lymphocyte cytosolic protein 1 (LCP1), which is integral to cell movement, has been observed to be upregulated in rats subjected to CIRI [116]. Recent studies have further supported this view and additionally identified lactylation modification of LCP1. Knocking down LCP1 or reducing LCP1 lactylation levels through glycolysis inhibition can rescue cell viability and prevent cell apoptosis [117]. Furthermore, a comparative analysis of calcium signaling pathway proteins regulated by Pan-Kla between CIRI and control groups in rats has revealed significant disparities. Notably, lactylation of the solute carrier family 25 members, Slc25a4 and Slc25a5, was exclusive to the CIRI group, while lactylation of voltage-dependent anion channel protein 1 (Vdac1) was detected solely in the control group [61]. These findings advocate for the hypothesis that calcium homeostasis in the brain, particularly under hypoxic conditions, is modulated by lactylation modifications, which in turn, have a substantial impact on the pathological mechanisms of CIRI.

ICH and SAH are another two common types of CVD, differentiated by the location of the bleeding. ICH is a type of hemorrhagic cerebrovascular disease characterized by localized bleeding in the brain due to non-traumatic rupture of blood vessels, closely linked to the process of neuronal ferroptosis [118]. The level of METTL3 lactylation is significantly increased along with its upregulation in the ICH cell model, resulting in reduced cell vitality. Lowering METTL3 decreases the m6A modification level of transferrin receptor (TFRC) and impairs its expression, effectively reducing intracellular iron concentration and preventing cellular ferroptosis [119]. SAH, a form of CVD, has been linked to lactylation modification, in addition to its occurrence in brain parenchyma. During the initial phase of SAH injury, astrocytes exhibit increased levels of lactate and Pan-Kla, coincident with a rise in BRD4 protein levels. In the C8D1A mouse astrocyte cell line, knockdown of BRD4 promotes the generation of the A1 subtype by obstructing lactylation modifications of H4K18. This leads to the release of numerous inflammatory factors and toxic substances into the surrounding microenvironment, threatening neuronal survival and resulting in impaired motor skills and behavioral changes observed in mouse behavioral tests [57].

Cerebral aneurysm (CA), a distinct variety of CVD, is closely associated with inflammatory processes triggered by oxidative stress within the cerebrovascular cellular milieu [120]. Under oxidative stress conditions, such as those induced by H2O2 treatment in vascular endothelial cells (VECs), the expression of LDHA is markedly diminished. Nevertheless, the cytoprotective effect of LDHA overexpression can be counteracted by vascular endothelial growth factor A (VEGFA). When vessels are in an oxidative stress state, the lactylation modification of LDHA induced by local hypoxia represents timely self-regulation and adaptation to the harsh environment. In CA, LDHA and VEGFA closely interact and coordinate with each other, influencing the pathophysiology of VECs under the backdrop of enhanced glycolysis, and providing new insights for management [121].

A variety of therapeutic strategies have been crafted to target lactylation modification pathways, offering promising avenues for treatment. A traditional approach involves the direct inhibition of lactylated proteins to achieve therapeutic effects, such as the inhibition of LCP1 and cGAS [110, 117]. From a metabolic standpoint, directly inhibiting LDHA or employing glycolysis inhibitors can curtail lactate production at its source, thereby mitigating CIRI and aiding in the restoration of cellular function [113, 117]. An alternative strategy involves modulating the activity of lactylation enzymes, such as the downregulation of the reader enzyme BRD4, to suppress lactylation processes. This approach can improve the motor and exploratory capabilities of mice with SAH, thereby enhancing neurological function [57].

Recognized as vital nutritional supporters and glycogen reservoirs for neurons, astrocytes play a pivotal role in maintaining neuronal health [122]. The phenomenon of mitochondrial transfer between neurons and astrocytes, as observed by Ni et al., is seen as one of the potential hopes that we can take advantage of for neuronal survival during ischemic brain injury [122]. Recently, Zhou et al. found that knockdown of low-density lipoprotein receptor-related protein 1 (LRP1) enhances cellular glucose uptake and glycolytic capability by increasing the density of glucose transporter type 1 (GLUT1) on the astrocyte membrane. This knockdown also induces lactylation at the K73 site in ADP-ribosylation factor 1 (ARF1), which affects vesicle assembly and induces mitochondrial loading abnormalities, thereby blocking the release of mitochondria from astrocytes, ultimately exacerbating the extent of brain infarction in mice. However, the innovative replacement of lysine at the K73 position in ARF1 can inhibit lactylation, foster the transfer of mitochondria from astrocytes to neurons, and effectively reduce the infarct area in mice [123]. It is important to note that, compared to astrocytes, the regulation of the multifunctional transmembrane receptor LRP1 on the surface of neurons has the opposite effect on glucose uptake capacity [124]. Therefore, it is necessary to explore the cellular differences in manipulating specific cell receptors and metabolic pathways.

In the realm of Traditional Chinese Medicine, ischemic CVD is often managed with Buyang Huanwu decoction (BHD), which has demonstrated favorable clinical efficacy [125]. Song et al. further validated the efficacy of BHD in rescuing the infarct area and blood-brain barrier permeability with the use of a rat model. They revealed that BHD diminishes lactylation modifications, including Pan-Kla and H3K18la, by restricting lactate synthesis, which leads to the normalization of apoptotic protease activating factor-1 (Apaf-1) transcription levels and ultimately promotes cellular survival [126].

**Fig. 3:**
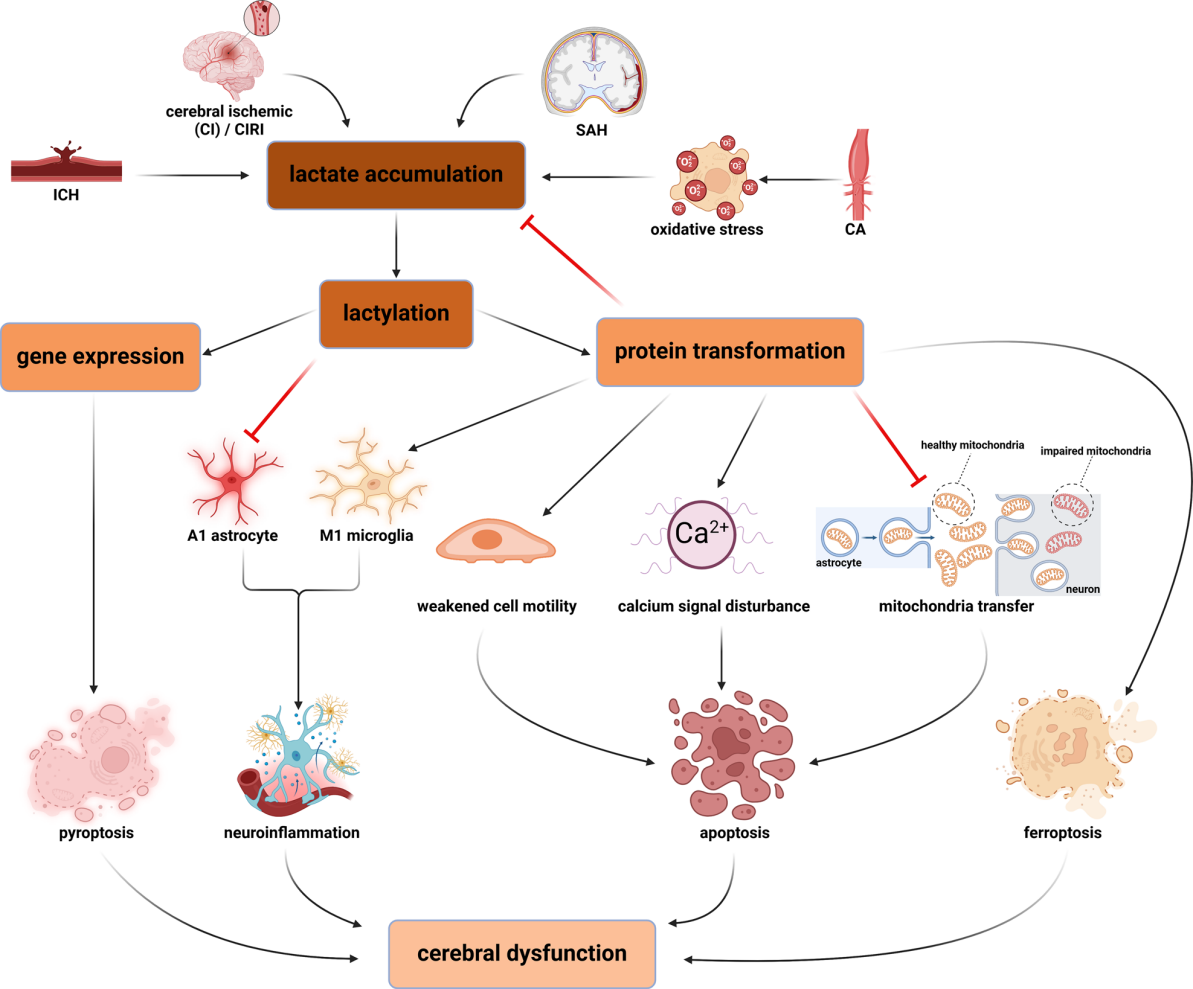
The regulatory role of lactylation in CVD pathology. The local tissue hypoxic damage caused by CI, ICH, and SAH, along with oxidative stress from CA, plays a critical role in the elevation of lactate levels. Lactate-mediated lactylation induces various cell death pathways and neuroinflammation through the regulation of gene expression and protein stability, ultimately leading to brain dysfunction. First, the initiation of pyroptosis is primarily attributed to the increased expression of the HMGB1 gene. The neuroinflammatory state involves the inflammatory activation of microglia and astrocytes, and lactylation modification can regulate the polarization of these two cell types to control neuroinflammation in the brain. Additionally, the onset of apoptosis is influenced by multiple factors. From the perspective of the cell itself, protein lactylation weakens cell motility and disrupts calcium signaling, ultimately triggering apoptosis. From the perspective of intercellular interactions, lactylation can disrupt mitochondrial transport between astrocytes and neurons, leading to neuronal death. Ferroptosis occurs due to the elevation of intracellular iron levels induced by protein lactylation.

The localized ischemic and hypoxic environment in brain tissue, along with the release of cytotoxic substances and the accumulation of inflammatory mediators due to tissue damage in CVD, creates favorable conditions for lactate accumulation, thereby supporting the rationale for lactylation research. Current studies involve various cell types, including microglia, astrocytes, and brain endothelial cells. Among these, the complex mechanisms of phenotypic transformation of astrocytes and microglia in CVD represent an important direction for future research. Although Wang et al. found that targeting cGAS and inhibiting lactylation modifications can promote the transition of microglia from the M1 phenotype to a repair phenotype [110], the addition of lactate has no effect on the expression of A2 astrocytes and only weakens M1 polarization process [57]. The complexity of this regulation requires further investigation for clarification. Furthermore, the pathogenic pathways mediated by lactylation in CVD are highly diverse, encompassing various forms such as apoptosis, necroptosis, ferroptosis, and inflammation. Therefore, the interconnections between different mechanisms should be emphasized, as this will enhance our understanding of metabolic changes and tissue damage in CVD (Fig. 3).

### Neuroinflammation

The dynamic activities and physiological shifts within microglia and astrocytes play a pivotal role in the initiation and escalation of neuroinflammation. While acute inflammation serves a beneficial function in tissue repair and the clearance of harmful elements following injury, its chronic manifestation is often implicated in the onset of neurodegenerative alterations [127]. The activation of microglia is typically bifurcated into two distinct phenotypes: the M1 phenotype, which is linked to pro-inflammatory activities and neurotoxic responses, and the M2 phenotype, which is engaged in tissue repair and neuroprotective processes. Astrocytes, in a similar vein, are classified into A1 and A2 subtypes, which bear functional resemblances to the M1 and M2 microglial phenotypes, respectively [128]. The transition of cellular metabolism towards glycolysis is closely related to the inflammatory activation of microglia, which can trigger neuroinflammation and neuronal cytotoxicity [129]. Under conditions of oxygen-glucose deprivation (OGD), astrocytes have also been observed to undergo abnormal activation, participating in mediating neuronal dysfunction through the secretion of inflammatory components [130]. Therefore, lactate and lactylation-related subtype transformation of astrocytes and microglia may play a crucial role in the onset and progression of neuroinflammation.

The aberrant inflammatory state triggered by cGAS lactylation-induced M1 polarization of microglia has been discussed above. The transcription factor NF-κB, integral to neuroinflammation, is intricately linked with the cGAS signaling pathway [131]. Recent studies have revealed its significant role in lactate-mediated inflammatory activation of microglia. In BV2 microglial cell lines subjected to lipopolysaccharide (LPS)-induced neuroinflammation, both lactylation-mediated inhibition of p53 activity and direct knockdown of p53 protein can disrupt the control of the NF-κB inflammatory signaling pathway, ultimately leading to abnormal expression of pro-inflammatory factors and M1 polarization of microglia [132]. In high-altitude cerebral edema (HACE) which is characterized by inflammation and hypoxia, proteins in the nucleosome remodeling and histone deacetylase (NuRD) complex of microglial is lactylated, which upregulates the expression of various pro-inflammatory factors in M1-type microglia and enhances NF-κB pathway activity, thereby exacerbating LPS-induced neuroinflammatory conditions [133]. For experimental autoimmune uveitis, abnormal activation of retinal microglia activated by YY1 lactylation also provides strong evidence that the subtype transitions of microglia are closely regulated by this PTM [38].

The inflammatory transitions of astrocytes exhibit complexity in tissue distribution and are regulated in a temporal and spatial dimension [134]. Emerging research on the lactylation of astrocytes suggests that this novel PTM is more inclined to direct cellular processes towards beneficial outcomes, rather than being a driver of inflammation. Given the close collaboration between astrocytes and neurons in energy metabolism [135], it is plausible that lactylation, triggered by the accumulation of lactate, may play a supportive role in bolstering neuronal energy metabolism and be a crucial factor in maintaining the metabolic equilibrium necessary for the proper functioning of neural networks.

What is worth mentioning is that the role of lactate in neuroinflammation mediated by microglia and astrocytes is dual-faceted, not acting merely as an initiator. Various studies have shown that lactylation can play a role in tissue repair and protection, showing potential as a therapeutic target. For example, under conditions of OGD, the local concentration of lactate increases, which triggers astrocyte responses by upregulating N-Myc downstream-regulated gene 2 (NDRG2) to alleviate the inflammatory state [136]. In the SAH mouse model, BRD4 can impede the generation of A1 subtype astrocytes by inducing Pan-Kla and H4K1la, thereby providing protection for neuronal survival [57]. For microglia, after spinal cord injury (SCI), the concentration of lactate increases, triggering H4K12 lactylation, which is involved in the repair and functional recovery of spinal cord injury [137]. Exercise treatment inhibited the overall expression of microglia in the hippocampus and cortex of the mice while upregulating M2 reparative microglia expression. Exogenous supplementation of lactate resulted in a comprehensive enhancement of the lactate/H3Kla/M2 phenotype conversion axis, indicating that lactylation acts as a protective mechanism, facilitating timely self-adjustment in response to inflammatory environments [47].

Based on existing research, we hypothesize that lactylation modifications have a time-specific impact on the activation state of microglia, similar to the lactate concentration sensing effect observed in AD. In the progression of neuroinflammation-related AD, the subtype transition of microglia exhibits a biphasic pattern [138]. During the acute phase of the disease, the body requires an inflammatory response to eliminate cellular damage, and now lactylation primarily supports the activation of pro-inflammatory microglia, inducing the first peak. Over time, the intrinsic regulatory mechanisms within microglia trigger protective responses that promote the transition to the M2 phenotype. The emergence of the second peak is related to the limitations of the M2 protective mechanisms induced by lactylation and the excessive deterioration of the disease state, as studies have shown that this protective response does not completely counteract the M1-induced inflammatory microenvironment—this transition begins with a delay and is relatively weak [47].

### Brain tumors

Gliomas are prevalent within the central nervous system, with GBM being particularly aggressive. GBM, which typically occurs in adults and the elderly [139], is notorious for its resistance to treatment and its grim prognosis, reflected in a 5-year survival rate of ~10%, and a <1% chance of surviving a decade post-diagnosis [140, 141]. The NF-κB pathway is widely expressed in various cell types within the central nervous system, supporting neuronal function [142]. However, this pathway can become detrimental when overactivated in GBM, promoting tumorigenesis and contributing to resistance to therapeutic interventions [143]. The high metabolic demands of malignant gliomas create a hypoxic tumor microenvironment (TME), triggering a shift in tissue metabolism towards glycolysis, which ultimately leads to elevated lactate levels [144]. To accommodate the tumor’s growth, there is an upregulation in the expression of angiogenic factors like vascular endothelial growth factor (VEGF), leading to the abnormal proliferation and expansion of tumor vasculature, a hallmark of high-grade gliomas [145]. Vasculogenic mimicry (VM) represents an innovative paradigm in tumor vascularization, diverging from the conventional angiogenesis that is endothelial cell-dependent. VM constitutes an alternative microcirculatory network, where tumor cells themselves contribute to the formation of blood vessels, playing a crucial role in various malignant diseases [146, 147]. The VEGFR2 present in situ within GBM is of significant importance in both angiogenesis and the construction and dynamic remodeling of VM [148]. In contrast to GBM, neuroblastoma (NB), which originates from the sympathetic nervous system, has a lower malignancy level and primarily affects children [149].

Recently, lactylation modifications have been observed to play an important role in tumor-associated signaling pathways in glioma. Wang et al. identified the lncRNA LINC01127 as a factor that exerts detrimental effects which is linked to lactylation modifications and LPS produced by the gut microbiota in colorectal cancer patients [150]. Subsequent reports have identified LINC01127 in the LN229 GBM cell line and in patient-derived HG7 cells. In the GBM microenvironment, increased lactate levels associated with the Warburg effect induced lactylation of histone H3, regulating LINC01127 transcriptional activity. LINC01127, in collaboration with Mitogen-activated protein kinase kinase kinase kinase 4 (MAP4K4), activates the JNK pathway to enhance the communication with the NF-κB pathway, culminating in the augmentation of the migratory capabilities and the self-renewal rate of glioma stem cells (GSCs) [151]. GSCs, as a highly specialized population of stem cells, play a crucial role in the progression and recurrence of GBM due to their ability to self-renew and continuously generate new tumor cells [152].

Aberrant activation of tumor signaling pathways leads to uncontrolled tumor proliferation, and the resultant unregulated cell growth induces the formation of new blood vessels locally to meet metabolic demands. Krüppel-like factors (KLFs) are pivotal in governing the biomechanical properties and homeostasis of the vascular wall, with their activity often modulated by a spectrum of PTMs [153]. Recent research has demonstrated that the encoding of P4-135aa peptide by pseudogene MAPK6P4 is responsible for the phosphorylation of the S238 site in KLF15 which targets LDHA to increase its expression. The ensuing lactylation modifications of VE-cadherin and VEGFR2 are closely linked to LDHA levels, which in turn exacerbate the progression of GBM [154].

In the context of high-grade NB, a defining characteristic is the overexpression of the oncogene MYCN [155]. Additionally, heightened levels of Hexokinase-2 (HK2) and LDHA have been observed [156]. Recent studies suggest that elevated HK3 expression in NB cells can intensify lactate metabolism, thereby fostering lactylation modifications that are mediated by intracellular lactate. This metabolic shift subsequently modulates the expression of CXCL14 (C-X-C Motif Chemokine Ligand 14), a protein implicated in the regulation of macrophage homeostasis, contributing to the polarization of M0-type tumor-associated macrophages (TAMs) towards the M2 phenotype, which is associated with enhanced motility and proliferative capacity of NB cells [157].

The pronounced malignancy of gliomas, particularly GBM, poses a significant therapeutic challenge worldwide. A strategic approach to treating GBM involves exploiting the nuances of DNA repair mechanisms to create a situation where the repair capacity is overwhelmed by the rate of damage, thereby enhancing the efficacy of therapies [158]. Recent research has shed light on the connection between homologous recombination (HR) repair and protein lactylation modifications, highlighting their impact on tumor progression and the development of therapeutic resistance [159, 160]. Yue et al. indicated that H3K9la binds to the transcription start site of LUC7L2, enhancing its transcriptional levels. LUC7L2 plays a role in the selective splicing that preserves the termination codon of MLH1, a key component of the mismatch repair (MMR) system. The impairment of MMR is a notable contributor to resistance against the chemotherapeutic agent Temozolomide (TMZ). Stiripentol, an LDH inhibitor, has been shown to significantly boost the effectiveness of TMZ through a mechanism that involves modulating lactylation [161]. Furthermore, Aldehyde dehydrogenase 1A3 (ALDH1A3) was observed to promote the tetramerization of PKM2, which facilitates lactate-induced lactylation of X-ray cross-complementing protein 1 (XRCC1) at the K247 site, directly leading to a diminished response of tumor cells to TMZ and radiation therapy. The small molecule compound D34-919, which targets the ALDH1A3-PKM2 interaction, has demonstrated clinically significant outcomes. This therapeutic strategy has been validated across various platforms, including in vitro studies, mouse models, and GBM organoids derived from patient tumor samples [162].

It is crucial to highlight that the resistance of GBM to treatments is not solely attributed to its enhanced DNA repair mechanisms. TMZ functions by methylating adenine and guanine at specific sites within the DNA sequence, resulting in the formation of three methylated products: N7-MeG, N3-MeA, and O6-meG. MMR system futilely attempts to remove thymine, which pairs optimally with O6-meG, yet fails to address the O6-meG lesion itself, leading to DNA strand breaks [163]. The efficacy of TMZ is intricately linked to the proper functioning of the MMR system, which explains why the downregulation of MLH1 can result in resistance to TMZ [161]. However, studies by Li et al. have demonstrated that heightened DNA repair activity mediated by XRCC1 is associated with resistance to TMZ as well as other radiotherapy and chemotherapy treatments. This association arises because XRCC1 plays a pivotal role in the base excision repair (BER) pathway, rather than in MMR [164]. BER enzymes are primarily targeted at removing N7-MeG and N3-MeA lesions, subsequently repairing the DNA through the collaborative action of DNA polymerase and DNA ligase, thus restoring the DNA strand to its original, non-damaged state [165]. In conclusion, when confronting drug resistance related to DNA repair, it is essential to focus on the specific DNA repair pathways implicated and the mechanisms by which therapeutic agents operate. Additionally, it is crucial to account for the interactions and synergies between various repair pathways, as the resolution of DNA damage in tumor cells often engages multiple mechanisms.

Although advancements have been made in targeting DNA repair mechanisms to treat GBM through chemotherapy, such conventional treatments also involving surgical interventions and radiotherapy have yet to yield consistently satisfactory outcomes [166]. Immunotherapy, which does not require drugs to traverse the blood-brain barrier, has emerged as an exceptionally promising treatment modality in recent years [167]. In GBM, a high proportion of indifferent immune cells and immunosuppressive non-lymphoid cells impair the immune system’s ability to combat tumor growth and invasion. The significance of Chimeric Antigen Receptor-T (CAR-T) cell therapy lies in its ability to enhance and equip the patient’s own T cells ex vivo to more effectively target tumor-associated antigens, thus rejuvenating weakened immune environments [168]. Sun et al. found that H3K18 lactylation positively regulates the transcription of CD39, CD73, and chemokine (C-C motif) receptor 8 (CCR8) to increase the proportion of Tregs, dampening immune activity. The application of oxamate by inhibiting lactate production can convert tumor-infiltrating lymphocyte and CAR-T cell phenotypes to a more potent state, maximizing tumor-killing effects and improving survival rates in GBM mouse models when combined with CAR-T [169]. TAMs, which include both microglia and monocyte-derived macrophages (MDMs), constitute the predominant immune cell population in GBM [170]. It has been reported that as GBM progresses to later stages of malignancy, MDMs increasingly replace microglia, becoming the dominant cell type and mediating immune suppression associated with the M2 phenotype. This shift is largely attributed to the expression of GLUT1 on the MDM cell surface, as MDMs lacking GLUT1 exhibit significantly diminished immune suppressive capabilities. Notably, lactylation related to GLUT-1 accumulates in the promoter region of the immunosuppressive factor interleukin-10 (IL-10), thereby significantly enhancing IL-10 transcription [48]. Moreover, lactate-mediated H3K18 lactylation positively regulates the transcription of tumor necrosis factor superfamily member 9 (TNFSF9), further linking lactate metabolism with M2 polarization events [171].

**Fig. 4:**
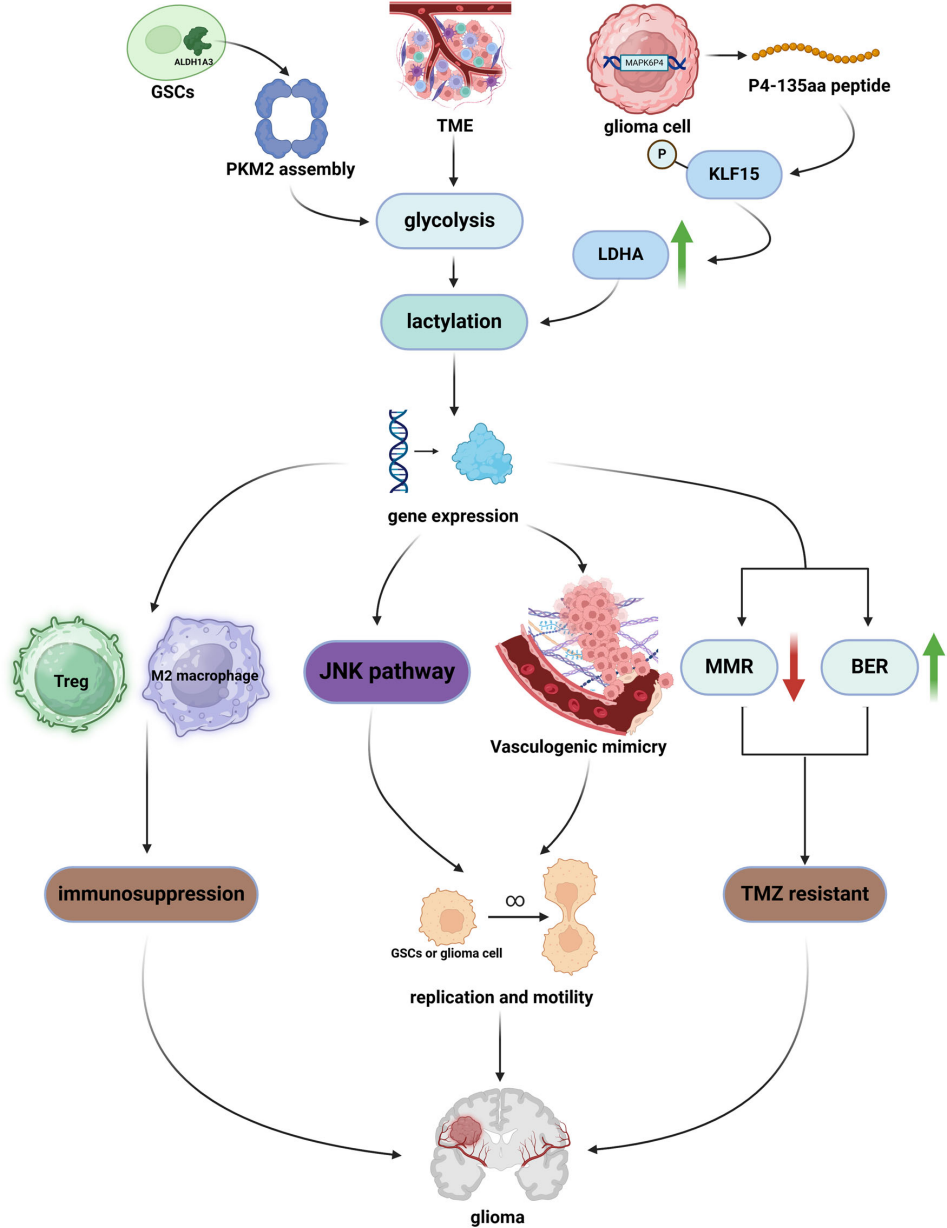
The regulatory role of lactylation in glioma pathology. The metabolic characteristics of the glioma microenvironment, along with changes in the activity of metabolic enzymes induced by abnormal gene expression, are key factors contributing to the increased lactylation modification in gliomas. Lactylation-mediated regulation of gene expression promotes glioma progression through various pathways. Firstly, enhanced interconnection of JNK and NF-κB pathways promotes the tumor cells’ inherent proliferative capacity, while abnormal vascular proliferation provides the necessary external conditions for the unlimited growth of tumor cells. Together, these factors synergistically enhance the cell’s motility and invasiveness. Additionally, lactate accumulation in advanced gliomas leads to an increase in the proportion of Treg cells and M2 macrophages in the tumor microenvironment, which mediates immune suppression and hinders the effectiveness of immunotherapy. Lactylation modification also contributes to tumor cell resistance to radiotherapy and chemotherapy by regulating DNA repair processes.

Developing effective therapeutic strategies that target the immune ecology in gliomas warrants further exploration. In the longitudinal immune landscape, gliomas are characterized in their early stages by proliferative and pro-inflammatory microglia [172]. Investigating the lactylation modifications of microglia during glioma progression should be a focus, as this can help rapidly control disease progression and effectively improve prognosis before deterioration occurs. Up to now, the choice of the specific treatment can be based on a lactylation scoring model, which reflected the intercellular activation and communication of immune cells within the TME, providing a new approach for assessing the disease status of glioma patients and guiding treatment strategies [173]. Furthermore, non-coding RNAs have been shown to be involved in the progression of gliomas [174]. Among these, both lncRNAs and pseudogenes have been found to be closely associated with lactylation in gliomas, while the unexplored domains of microRNAs and circRNAs are promising and intriguing (Fig. 4).

### Neurodevelopmental diseases

The dynamics of energy metabolism and histone modification are pivotal during neurodevelopment. Maturation from neural stem cells (NSCs) to fully differentiated neurons is characterized by a metabolic shift from predominant glycolysis to OXPHOS, which is instrumental in guiding the developmental trajectory [175]. During the posterior embryonic axial development in mice and chicks, a glycolytic gradient is observed, which modulates the expression of various signaling pathways, thereby regulating developmental processes [176]. This spatiotemporal heterogeneity in glycolysis indicates that fluctuations in lactate levels during the early stages of neurodevelopment may exert significant regulatory effects. Furthermore, neural crest cells (NCCs), which are poised to undergo epithelial-mesenchymal transition, also demonstrate heightened glycolytic activity and lactate accumulation. These metabolic changes are crucial for NCCs’ migration and the determination of their cellular identities [177]. Recently, the impact of lactate-mediated lactylation in this process has also been unveiled.

In terms of the overall regulation of neurodevelopment, Dai et al. demonstrated that the levels of H3K18 lactylation gradually decrease in P19 embryonic carcinoma (EC) cells as neurogenesis and differentiation progress. Despite H3K18la being predominantly found in transcriptionally inert regions like introns, the optimal functioning of transcriptional start sites necessitates a complex interplay of various PTMs, among which lactylation is a key player [54].

Within the nascent phases of neurodevelopment, NCCs ramp up their production of lactate through LDHA/B, steering their metabolism toward glycolysis. This metabolic shift promotes lactylation of certain nuclear genes related to cell adhesion and migration, such as SOX10, thereby enhancing the migratory capacity of NCCs [178]. The migration of NCCs plays an indispensable role in the development of midfacial structures, and the precise control of tissue growth and fusion is paramount to prevent significant developmental anomalies [179]. In an innovative approach, a mouse model was engineered to express bone morphogenetic protein (BMP) receptors ACVR1 constitutively in the neural crest region, thereby enhancing BMP/Smad signaling. The intensification of the BMP pathway was observed to downregulate the expression of pivotal glycolytic genes in a p53-dependent fashion, consequently inhibiting Pan-Kla and H3K18la. As a result, the expression of epigenetic downstream targets, notably Pdgfra—a gene essential for facial development—was impeded, leading to severe defects in midline facial development in the majority of the mice [180].

As neurodevelopment progresses, the intricate regulatory network of histone PTMs becomes increasingly vital in sculpting neuronal formation. Genes that exhibit increased enrichment of H3K9cr and H3K18la are predominantly involved in neuronal differentiation and maturation. These modifications, in conjunction with H3K9ac, orchestrate the intricate processes governing neuronal cell fate [54]. Beyond the realm of neurons, the development and functionality of microglia and astrocytes are also influenced by the metabolic landscape, which is associated with defects in the glycolytic pathway mediated by the loss of BTB and CNC homology 1 (Bach1) [181]. The role of Bach1 in glycolytic enzymes and lactate metabolism has been well documented [182]. Bach1 induces the accumulation of lactate-mediated H4K12 lactylation at the promoter region of LRRC15, targeting CD248/GP130/JAK-STAT3 axis to influence the development of astrocytes [181].

### Mental disorders

Anxiety is acknowledged as the most widespread condition among mental disorders, often occurring alongside depression [183]. Schizophrenia (SCZ), another prevalent mental disorder, has an incidence rate of ~0.5% in China [184]. The hallmark of epilepsy is the imbalance in brain energy metabolism. During seizures, the excessive neuronal excitability is closely linked to the energy support provided by astrocytes to neurons, known as the ANLS [185]. Research has established a strong correlation between lactate levels and both the precision of synaptic networks and neuronal excitability [186]. Enhanced glycolysis or alterations in brain energy metabolism, marked by lactate accumulation, have been detected in major depressive disorder [187], anxiety disorders [188], SCZ [189], and epilepsy [190]. Recently, chronic stress and increased neuronal excitability have been identified as pivotal factors that stimulate lactylation in various neural cells of the brain, especially in the medial prefrontal cortex (mPFC), a region considered to be closely related to anxiety and depression-like behaviors in mice [191]. This represents the first comprehensive systemic analysis of lactylation modifications in the brain, providing a solid foundation for further exploration into the role of lactylation in the spectrum of psychiatric disorders.

Recent studies have uncovered that regular physical activity can modulate lactate levels in the mPFC of mice, concurrently augmenting lactylation at the K885 residue of synaptosome-associated protein 91 (SNAP91). This specific lactylation event is crucial for maintaining the activity of neuronal networks and for stabilizing synaptic structures, highlighting its essential role as a mediator in the anxiolytic effects of exercise [192]. These findings, in conjunction with prior studies [193], corroborate the mPFC’s centrality in the development of anxiety and depression, underscoring the necessity for a deeper exploration into the regulatory dynamics of lactylation modifications within this region. Moreover, in mouse and cellular models of SCZ constructed by MK801, an accelerated glycolytic metabolism accompanied by high lactate accumulation has been observed, resulting in increased Pan-Kla, H3K9la, and H3K18la levels. Impairments in neuronal survival mediated by HMGB1 are believed to be closely associated with the glycolytic pathway, as evidenced by the mitigation of these adverse effects through treatment with the glycolysis inhibitor 2-DG [194]. Furthermore, 2-DG-mediated inhibition of glycolysis effectively reverses various electrophysiological alterations induced by epilepsy, suggesting that targeting lactate metabolism pathways could be a promising approach for the development of novel antiepileptic drugs [195] (Supplemental Table 2).

## Future perspectives

Lactate, a key participant in anaerobic metabolism, has increasingly become the focus of research, particularly regarding its bioavailability and production in various pathological and physiological states. The phenomenon of lactylation represents a burgeoning field, indicating a more advanced role of lactate in metabolic pathways associated with disease. Based on existing research, the accumulation of lactate triggered by various biological processes is an important prerequisite for the occurrence of lactylation modifications. However, it remains unclear whether lactylation is merely a natural consequence of elevated lactate levels or if it requires specific regulatory factors for precise modulation. Future research needs to continue elucidating the specific mechanisms involved, as this is crucial for guiding us toward a deeper understanding and the development of more effective treatment strategies.

It is noteworthy that the role of LDH in lactylation should be reevaluated. Immunoprecipitation has revealed that LDHA can directly bind to proteins and participate in lactylation modifications.. Silencing LDHA results in a significant reduction in the lactylation levels of its target proteins, with no observed effect on LDHA’s role in metabolism. This suggests that LDHA may not function as a metabolic enzyme within the conventional context of glycolytic pathways, but rather plays a direct regulatory role in the addition of lactylation modifications [154]. Additionally, despite the strong correlation between LDH activity and adverse prognosis as well as malignancy in NB cells, sometimes the downregulation of LDHA and LDHB does not impact cellular glycolytic activity or lactate concentration [196]. This observation suggests a potentially different mechanism underlying the suppressive effects of LDH inhibition in NB, it may extend beyond its recognized metabolic functions in tumorigenesis.

Here, we offer a review of the latest research developments concerning lactylation in the context of brain diseases. Overall, research into the regulatory role of lactylation in brain dysfunction remains largely confined to pathogenic pathways, with limited exploration of drug targets and therapeutic prospects. Although some studies have found that targeting lactylation-related regulatory axes can improve the behavioral activities or physiological states of mice, these are few in number and require broader research support. Notably, the efficacy of the small molecule compound D34-919 has been validated in organoid models of gliomas [162]. Organoids, due to their capacity to replicate the original tumor characteristics and immune microenvironment, are recognized as powerful tools for studying cancer immunotherapy [197]. In future investigations into the regulation of lactylation in brain tumors, incorporating organoid models should be considered.

Furthermore, given that lactylation regulatory enzymes often exhibit ACT properties, the therapeutic prospects for targeting these enzymes remain concerning. Research has already revealed a competitive relationship between acetylation and lactylation modifications at the H3K18 site, and inhibition of erasers has not yielded the anticipated restoration of lactylation levels [50]. Cui and colleagues have also expressed similar concerns, positing that p300 is not only one of the most crucial ACTs but also possesses a multitude of other cellular functions [28]. The competition between different protein acylations, particularly lactylation and acetylation, is a key consideration when exploring treatment strategies for CNS diseases. Although this competitive phenomenon has not yet been observed in the brain, it may be a significant reason for the poor efficacy and side effects of many therapeutic approaches, warranting further investigation. Specifically targeting lactylation without affecting other acylations is crucial for achieving therapeutic effects and avoiding unintended side effects. A more comprehensive understanding of the relationship between lactylation and other PTMs, as well as the regulatory strategies for protein lactylation, will aid in developing new therapeutic ideas and expanding the treatment potential of lactylation modifications in CNS diseases.

## Conclusion

In conclusion, this review demonstrates that lactate and lactylation modifications, due to their involvement in critical brain metabolic pathways and their regulation of various gene transcription and protein functions, play a crucial regulatory role in the context of brain diseases. Writers, erasers, and readers contribute to maintaining the dynamic balance of enzyme-catalyzed lactylation modifications, which holds significant implications for the progression of brain dysfunction.

## Supplementary information

Supplemental Table
