# Supplementary material for: The role of protein lactylation in brain health and disease: current advances and future directions
Source: Cell Death Discov. 2025 Apr 30;11:213. doi: 10.1038/s41420-025-02408-w (PMC12043837; doi:10.1038/s41420-025-02408-w)
Supplement: Supplementary file 1 — Supplemental Table [file 41420_2025_2408_MOESM1_ESM.docx]

Supplemental Table 1: Writers, erasers, and readers

| Enzyme | PMID |
| --- | --- |
| P300 | 38874057，38679071，38852200，38711083，38493493，38467179，38512058，38769664，37443257，37085894 |
| CBP | 38128537，37172660，36735787 |
| MOF | 39102921，38359291 |
| HBO1 | 38670996 |
| TIP60 | 38961290 |
| GCN5 | 36268709 |
| GNAT13 | 37669396 |
| YiaC | 36333310 |
| AARS | 39322678，**37863889，38512451，38163844，38653238，39322678** |
| ATAT1 | 38879723 |
| HDAC1 | 38874057，38467179，35500056，35735108，35044827 |
| HDAC2 | 38711083，35500056，35735108，35044827 |
| HDAC3 | 38874057，38961290，35500056，35735108，35044827 |
| SIRT1 | 38511243，37443257，35500056，35815577 |
| SIRT2 | 35500056，35815577 |
| SIRT3 | 36896611，37720100，35500056，35815577 |
| CobB | 36333310 |
| Brg1 | 38512058 |

The lactylation writers, erasers, and readers identified to date are listed in Supplemental Table 1. For each enzyme, we have provided the corresponding research literature PMID numbers, which can be directly searched on PubMed for a better understanding of the role these regulatory enzymes play in lactylation modification.

Supplemental Table 2: Lactylation and disease-associated pathway

| Pathogenic pathways or therapeutic pathways | Lactylation site | Downstream function | Disease | Lactylation enzyme | Effects of lactylation | PMID: |
| --- | --- | --- | --- | --- | --- | --- |
| P. Gingivalis/msRNA/lactylation/Aβ accumulation | Histone | Unknown | AD | Unknown | Harmful | 37320870 |
| EPB41L4A-AS1/lactylation/autophagy/Aβ removal | Histone | Promote gene expression | AD | GCN5L2 (writer) | Beneficial | 38704365 |
| IDH3β↓/lactylation/PAX6 (positive feedback) | H4K12, H4K8 and H3K18 | Promote gene expression | AD | Unknown | Harmful | 38679634 |
| Glycolysis/lactylation/PKM2 (positive feedback) | H4K12 | Promote gene expression | AD | Unknown | Harmful | 35303422 |
| Lactylation/NFκB pathway/SASP IL-6 and IL-8 | H3K18 | Promote gene expression | AD | Unknown | Harmful | 37697347 |
| Lactylation/M2 microglia/Aβ↓ | Histone 3 | Promote gene expression | AD | P300 (writer) | Beneficial | 37978517 |
| Glycolysis/lactylation/SLC7A11/microglia activation | H3K9 | Promote gene expression | PD | P300/CBP(writer) | Harmful | [39753581](https://pubmed.ncbi.nlm.nih.gov/39753581/) |
| LDHA/lactylation/HMGB1/pyroptosis | H3K18 | Promote gene expression | CI | Unknown | Harmful | 36870018 |
| Lactylation/calcium disturbance/apoptosis | Slc25a4 and Slc25a5 | Change the physicochemical property of protein | CI | Unknown | Harmful | 36030297 |
| Glycolysis/lactylation/apoptosis | LCP1 | Change the physicochemical property of protein | CI | Unknown | Harmful | 36574182 |
| Glycolysis/lactylation/M1 microglia↑ | cGAS | Change the physicochemical property of protein | CI | Unknown | Harmful | 38246965 |
| LRP1/glycolysis/lactylation/mitochondria transfer↓ | ARF1 | Change the physicochemical property of protein | CI | Unknown | Harmful | 38906140 |
| Glycolysis/lactylation/Apaf-1/apoptosis | Pan-K, H3K18 | Promote gene expression | CI | Unknown | Harmful | 39029715 |
| Lactylation/m^6^A modification of TFRC/ferroptosis | METTL3 | Change the physicochemical property of protein | ICH | Unknown | Harmful | 37105375 |
| BRD4/lactylation/A1 astrocyte↓/recovery of neuron | H4K18 | Unknown | ICH | BRD4 (reader) | Beneficial | 39080649 |
| Oxidative stress/glycolysis/lactylation/VEC recovery | LDHA | Unknown | CA | Unknown | Beneficial | 38829380 |
| LPS/lactylation/NF-κB pathway/M1 microglia↑ | p53 | Change the physicochemical property of protein | Neuroinflammation | Unknown | Harmful | 38908518 |
| LPS and hypoxia/lactylation and NF-κB pathway/M1 microglia | NuRD complex | Change the physicochemical property of protein | Neuroinflammation | Unknown | Harmful | 39054523 |
| Glycolysis/lactylation/LINC01127/MAP4K4/JNK pathway/NF-κB pathway/GSCs motility and renewal | Histone 3 | Promote gene expression | GBM | Unknown | Harmful | 38084701 |
| lactate/lactylation/CD39, CD73 and CCR8↑ /immunosupression | H3K18 | Promote gene expression | GBM | Unknown | Harmful | 37770937 |
| Glycolysis/lactylation/IL-10/immunosupression | Histone | Promote gene expression | GBM | P300 (writer) | Harmful | 38703775 |
| MAPK6P4/P4-135aa/KLF15 /LDHA/lactylation/VM, tumor cell motility and renewal | VEGFR2 and VE-cadherin | Promote gene expression | GBM | LDHA (writer) | Harmful | 37853052 |
| Glycolysis/lactylation/LUC7L2/MLH1↓/TMZ resistance | H3K9 | Promote gene expression | GBM | Unknown | Harmful | 38477507 |
| HK3/lactate/lactylation/CXCL14/M2 TAM/tumor cell motility and renewal | Histone | Promote gene expression | NB | Unknown | Harmful | 38714539 |
| ALDH1A3/PKM2/glycolysis/lactylation/XRCC1/TMZ and radiotherapy resistance | XRCC1 | Change the physicochemical property of protein | GBM | Unknown | Harmful | 39111285 |
| Hypoxia/glycolysis/lactylation/TNFSF9/M2 macrophage | H3K18 | Promote gene expression | Glioma | Unknown | Harmful | 39010835 |
| ACVR1/BMP pathway/p53/glycolysis↓/lactylation↓/ Pdgfra↓/Craniofacial anomalies | Pan-K, H3K18 | Promote gene expression | Craniofacial anomaly | Unknown | Beneficial | 38466355 |
| Physical exercise/lactate/lactylation/synaptic formation and neuronal activity/anxiety↓ | SNAP91 | Change the physicochemical property of protein | Anxiety | Unknown | Beneficial | 39163863 |
| Glycolysis/lactylation/HMGB1/neuronal death | Pan-K, H3K9 and H3K18 | Promote gene expression | SCZ | Unknown | Harmful | 37207839 |

Here, we summarize all key pathogenic axis associated with brain disorders, listing the targets of lactylation, downstream effects, corresponding disease types, and whether lactylation-regulating enzymes are involved. You can quickly access the relevant information by searching the provided PMID numbers.
